# Complete plastid genome of *Gentiana zollingeri* Fawcett (Gentianaceae) and phylogenetic analysis

**DOI:** 10.1080/23802359.2022.2152644

**Published:** 2022-12-09

**Authors:** Jing Yu, Pengcheng Fu, Shanshan Sun

**Affiliations:** a Affiliated Hospital of Zhengzhou University, Zhengzhou, China; b School of Life Science, Luoyang Normal University, Luoyang, China

**Keywords:** *Gentiana zollingeri*, plasomte, phylogenetic analysis

## Abstract

*Gentiana zollingeri* Fawcett (Gentianaceae) belongs to the most species-rich section, *Chondrophyllae*, in the Gentianaceae, but its phylogenetic relationship with other members of this section is unclear. To confirm its phylogenetic position, the complete plastid genome of *G. zollingeri* was determined and analyzed. The plastome was sequenced using the Illumina HiSeq platform, assembled with GetOrganelle, and annotated with GeSeq. The genome is circular with a length of 130,762 bp. It contains a large single-copy (LSC) region of 74,236 bp, a small single-copy (SSC) region of 10,598 bp, and two inverted repeat (IR) regions of 22,964 bp each. The plastome of *G. zollingeri* shows considerable structural differences from those of other *Gentiana* plastomes, such as the absence of the *ndh* gene. In phylogenetic analyses, section *Chondrophyllae*, including *G. zollingeri* and its sisters, formed a long branch sistering with section *Cruciata*. The plastome sequence described here represents an important contribution to phylogenetic and evolutionary studies on *Gentiana*.

*Gentiana* is an alpine genus of about 360 species that are distributed worldwide, with the Qinghai-Tibet Plateau as the center of its distribution and diversity (Ho and Liu 2001; Favre et al. 2016). Out of the 13 sections in *Gentiana*, section *Chondrophyllae* Bunge is the most species-rich taxon, containing about 180 species (Ho and Liu 2001; Favre et al. 2020). Although many *Gentiana* plastome sequences are available, genome data are very limited for members of section *Chondrophyllae* (Fu et al. 2021). *Gentiana zollingeri* Fawcett, 1883 (Figure 1), having value in Chinese traditional medicine, belongs to section *Chondrophyllae* series *Fastigiatae* T.N. Ho, and is widely distributed in China, Russia, Korea, and Japan (Ho and Liu 2001).

**Figure 1.**
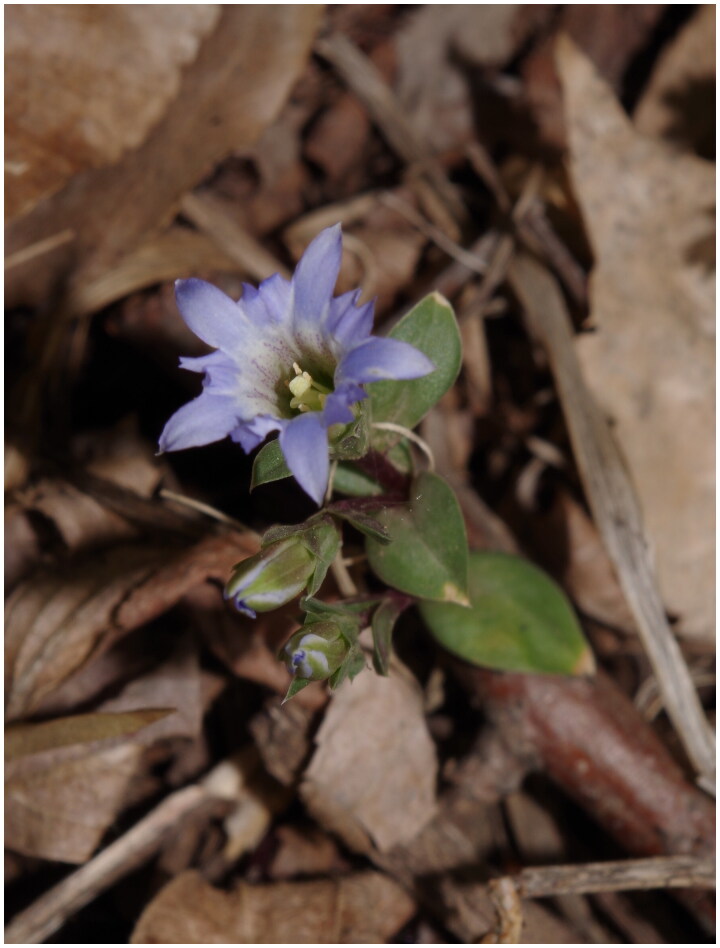
*Gentiana zollingeri. Gentiana zollingeri* is an annual, 3–6 cm tall. Stems purple; flowers few to many; corolla pale blue; stamens equal. Photographs by Pan Li.

Herein, we report the complete plastome of *G. zollingeri* (MZ934753) and its main characteristics. One *G. zollingeri* individual was collected from Liangjiatai Town, Hebei Province, China (40°46′N, 118°48′E). The voucher specimen was deposited at the Herbarium of Luoyang Normal University (Bin Cai, 987869364@qq.com) under the voucher number LP161491, and was identified by Dr. Pengcheng Fu. We extracted total DNA using a Dzup plant genomic DNA extraction kit (Sangon, Shanghai, China). The fragmented genomic DNA was sequenced using the Illumina HiSeq 2500 platform (Novogene, Tianjin, China), yielding 2 Gb clean data consisting of paired-end reads. The plastome was assembled using GetOrganelle version 1.7.1 (Jin et al. 2020) and annotated using GeSeq (Tillich et al. 2017) with the default parameters. The genome map was drawn using CPGview (http://www.1kmpg.cn/cpgview) with the GenBank accession number (MZ934753). The plastome was compared with those of species from the main clades of *Gentiana* (Zhou et al. 2018; Sun et al. 2018; Sun, Wang, et al. 2019; Fu et al. 2021), and structural changes were detected using mVISTA (Frazer et al. 2004). To confirm the phylogenetic position of *G. zollingeri*, the shared protein-coding genes in plastomes of all main *Gentiana* clades were extracted and then aligned using MAFFT version 7 (Katoh and Standley 2013) in PhyloSuite version 1.1.15 (Zhang et al. 2020). Using concatenated data, maximum likelihood phylogenetic analyses were conducted using IQ-TREE version 1.6.12 (Nguyen et al. 2015) with 1000 replicates. The substitution model was chosen using ModelFinder 2 (Kalyaanamoorthy et al. 2017). *Metagentiana rhodantha* (GenBank accession no. MN199153, Fu et al. 2021) was used as the outgroup.

The complete plastome of *G. zollingeri* is 130,762 bp in length. The LSC and SSC regions consist of 74,236 and 10,598 bp, respectively, and there are two IRs of 22,964 bp each. A total of 122 genes are annotated, consisting of 80 protein-coding genes, 34 tRNA genes, and eight rRNA genes (Figure 2). Comparative analyses indicate that the *ndh* complex in the plastome of *G. zollingeri* has undergone numerous gene loss (*ndh*A, *ndh*C, *ndh*E, *ndh*F, *ndh*G, *ndh*I, *ndh*J and *ndh*K) and pseudogenization events (*ndh*B, *ndh*D and *ndh*H). The absence of the *ndh* complex is also detected in the *Gentiana* sections *Chondrophyllae* (Fu et al. 2021) and *Kudoa* (Sun et al. 2018).

**Figure 2.**
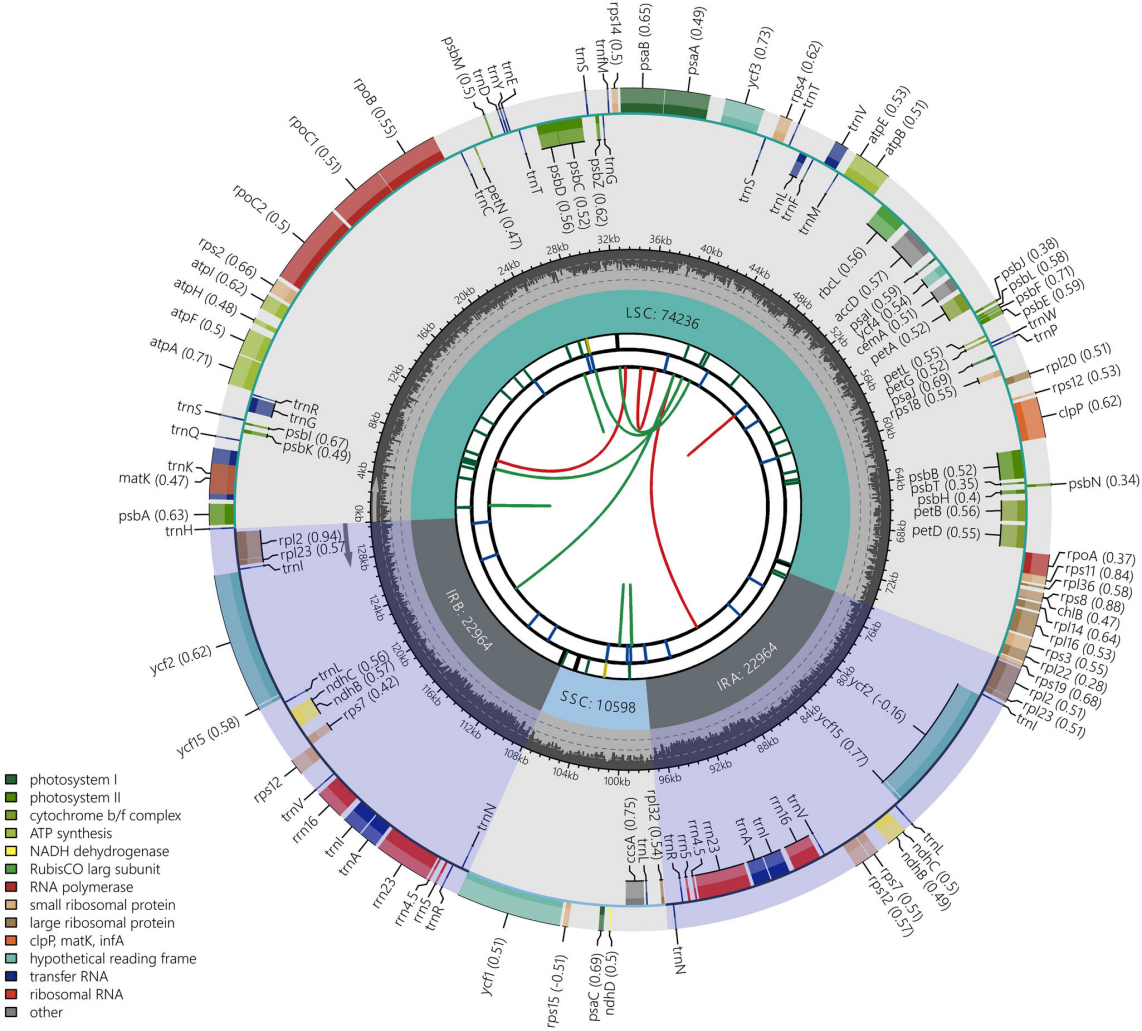
Schematic map of overall features of the chloroplast genome of *Gentiana zollingeri*. Graphic showing features of the *Gentiana zollingeri* plastome was generated using CPGview (http://www.1kmpg.cn/cpgview). The map contains seven circles. From center outwards, first circle shows distributed repeats connected with red (forward direction) and green (reverse direction) arcs. Second circle shows tandem repeats marked with short bars. Third circle shows microsatellite sequences as short bars.Fourth circle shows sizes of long single copy (LSC) and short single copy (SSC) regions. Fifth circle shows two inverted repeat (IR) regions: IRA and IRB. Sixth circle shows GC contents along plastome. Seventh circle shows genes marked with different colors according to their functional groups.

The phylogenetic relationships among all main *Gentiana* sections are fully supported. The phylogenetic analysis shows that *G. zollingeri* is more closely related to *G. aristata* and *G. producta* than to *G. leucomelaena*, *G. haynaldii*, and *G. cuneibarba* (Figure 3), indicating that the series *Humiles* (containing *G. aristata* and *G. leucomelaena*) and *Dolichocarpa* (containing *G. producta* and *G. haynaldii*) in section *Chondrophyllae* are not monophyletic. Notably, *G. filistyla* forms a sister group with section *Kudoa* rather than with section *Isomeria* to which it belongs (Ho and Liu 2001). This finding is also supported by nuclear data (Favre et al. 2020). The plastome sequence of *G. zollingeri* reported here provides new molecular data that illuminate aspects of the phylogenetic and molecular evolution of *Gentiana*, particularly the most species-rich section, *Chondrophyllae*.

**Figure 3.**
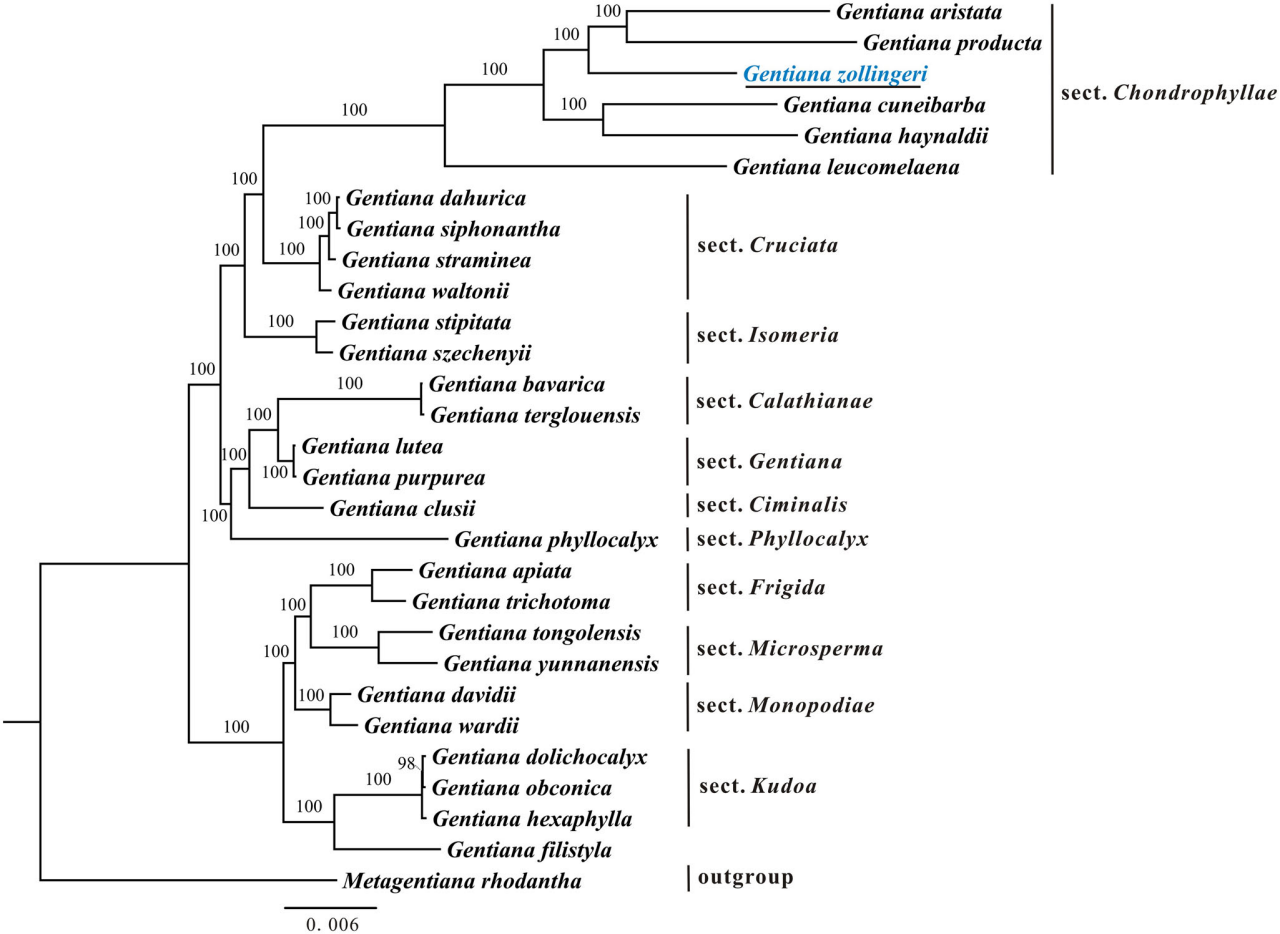
Maximal likelihood tree of *Gentiana* based on protein-coding genes in the plastome. Numbers above branches present bootstrap support. Species shown in blue is the newly sequenced in this study. The following sequences were used: *G. aristata* MN234139 (Fu et al. 2021), *G. producta* MN199163 (Fu et al. 2021), *G. cuneibarba* MN199137 (Fu et al. 2021), *G. haynaldii* MN234137 (Fu et al. 2021), *G. leucomelaena* MT905404 (Ya et al. 2021), *G. dahurica* MH261259 (Zhou et al. 2018), *G. siphonantha* MH261260 (Fu et al. 2021), *G. straminea* KJ657732 (Ni et al. 2016), *G. waltonii* MK780032 (unpublished), *G. stipitata* MG192309 (Sun et al. 2018), *G. szechenyii* MN199158 (Fu et al. 2021), *G.bavarica* MN199162 (Fu et al. 2021), *G. terglouensis* MN199132 (Fu et al. 2021), *G. lutea* MN199129 (Fu et al. 2021), *G. purpurea* MN199146 (Fu et al. 2021), *G. clusii* MN199142 (Fu et al. 2021), *G. phyllocalyx* MN199163 (Fu et al. 2021), *G. apiata* MK317975 (She et al. 2019), *G. trichotoma* MN089577 (Sun, Wang, et al. 2019), *G. tongolensis* MG251985 (Sun, Zhou, et al. 2019), *G. yunnanensis* MN199140 (Fu et al. 2021), *G. davidii* MN199156 (Fu et al. 2021), *G. wardii* MN234136 (Fu et al. 2021), *G. dolichocalyx* MN199161 (Fu et al. 2021), *G. obconica* MG192306 (Sun et al. 2018), *G. hexaphylla* MG192305 (Sun et al. 2018), *G. filistyla* MN199134 (Ya and Miao 2020), *Metagentiana rhodantha* MN199153 (Fu et al. 2021).

## Data Availability

The genome sequence data that support the findings of this study are openly available in GenBank of NCBI at [https://www.ncbi.nlm.nih.gov] (https://www.ncbi.nlm.nih.gov/) under the accession no. MZ934753. The associated BioProject, SRA, and Bio-Sample numbers are PRJNA758493, SRR15661475, and SAMN21028202, respectively.
